# Precision medicine in myeloma demands precision imaging: is whole body MRI the solution?

**DOI:** 10.1186/s40644-026-01017-9

**Published:** 2026-03-11

**Authors:** Christina Messiou, Martin Kaiser

**Affiliations:** 1 The Royal Marsden Hospital, London, UK; 2 The Institute of Cancer Research, London, UK

## Abstract

Outcomes for patients with multiple myeloma have improved substantially over the past two decades, with median overall survival more than doubling in many cohorts. Management of smouldering myeloma has also shifted, with randomised trials demonstrating that early intervention in high-risk disease delays progression and improves survival compared with observation alone. Imaging diagnostics must therefore evolve in parallel to provide minimally invasive, sensitive and quantitative insights into disease for patients who will live with myeloma for many years. Here we review the latest evidence for WB-MRI in myeloma, outline current guideline recommendations, and consider future directions.

## Introduction

Multiple myeloma (MM) is a heterogeneous, systemic cancer that can manifest anywhere, often in multiple locations across, and sometimes outside, the bone marrow. Recent substantial progress in improving survival reflects major advances in systemic therapy, supportive care, and earlier, more accurate diagnosis. Novel agents—including proteasome inhibitors, immunomodulatory drugs, monoclonal antibodies, bispecific T-cell engagers and CAR-T cell therapies—have enabled deeper and more durable responses, particularly when used in combination and in risk-adapted treatment strategies. However, even when patients achieve deep responses small numbers of residual malignant plasma cells usually persist. Current treatments, while highly effective, rarely eradicate disease permanently. In most patients, MM is not considered curable, but it is increasingly treatable and can be controlled for many years. Imaging plays a central role in diagnosis, but it must now contribute to risk stratification, prognostication and response assessment.

## WB-MRI: rationale and evidence base

Historically, imaging contributed to myeloma diagnosis through the detection of lytic bone destruction on plain film/skeletal survey or CT. However, with the introduction of biological therapies with fewer side effects, the International Myeloma Working Group (IMWG) acknowledged that waiting for irreversible bone damage before starting therapy was no longer justifiable. The updated diagnostic criteria therefore introduced a positive MRI, defined as the presence of one of more unequivocal focal lesions >5 mm, as one of the key diagnostic criteria for symptomatic myeloma, warranting systemic therapy [1]. This shift from cortical bone to bone marrow imaging enables myeloma diagnosis ahead of bone destruction.

Consequently, a multidisciplinary consensus group convened to establish standardised WB-MRI acquisition. The MY-RADS protocol consists of anatomical and functional imaging: sagittal spine, axial diffusion weighted and Dixon imaging from skull vertex to upper thighs [2]. The complement of sequences and coverage permits highly sensitive disease detection alongside detection of skeletal events and extramedullary disease. Over the past 10 years evidence has positioned WB-MRI as the most sensitive imaging modality for focal disease detection and although not part of the diagnostic criteria is also sensitive to detection of diffuse infiltration which is thought to be associated with higher risk disease.

IMWG guidance positions highly sensitive WB-MRI for myeloma diagnosis if CT or FDG PET/CT are negative but also for annual surveillance of patients with smouldering multiple myeloma (SMM) for a period of 5 years [3]. However, a holistic review of the evidence accumulated by the UK National Institute for Health and Care Excellence including sensitivity, specificity, quality of life and health economics led to national guidance that WB-MRI should be used first line for all patients with a suspected diagnosis of MM [4]. This guidance together with initiatives to support training have seen the use of WB MRI in the UK grow from 27% in 2018 to 69.2% [5] in 2024 with a global survey also indicating increasing adoption [6]. System-level and financial differences have hindered adoption in the US although the gap is narrowing with many elite US myeloma centres forging ahead by negotiating insurance pre-authorisations or billing multiple regional MRIs.

Although the initial drivers for WB-MRI were centred around diagnosis, more recently risk stratification in smouldering myeloma has been a major catalyst where the sensitivity of WB-MRI is driving treatment decisions. WB-MRI can reclassify SMM into early symptomatic MM and identify patients whose laboratory measures underestimate disease burden. Conversely it can also provide reassurance that surveillance is safe. The growth of curative-intent trials and therapy in SMM will increasingly depend upon precision diagnostics including WB-MRI.

It is well recognised that WB-MRI is complimentary to marrow sampling for diagnosis and assessment of disease burden as marrow trephine is prone to sampling error in this heterogeneously distributed disease [7]. This complementarity also plays out in the setting of minimal residual disease (MRD) where patients with residual disease on WB-MRI have been shown to have lower progression free and overall survival [8, 9] with independent information to matched serological response and minimal residual disease.

## Future directions

WB-MRI is now established as a clinical trials ready modality with a track record in the multicentre setting across the 3 main MRI vendors [10]. This will become increasingly relevant in a population of patients with increased life expectancy where the effects of radiation from non-MRI examinations become more challenging to justify.

The evolution of WB-MRI in myeloma imaging will be shaped by technological, clinical, and operational advances. Faster acquisition strategies [11] are reducing scan times and improving patient tolerance. AI-driven automated reporting tools could reduce reading times and improve diagnostic consistency, addressing one of the barriers to widespread implementation. Quantitative WB-MRI biomarkers such as ADC values, total diffusion volume (TDV), and fat-fraction measurements unlocked by automated segmentations hold promise as objective measures of tumour burden and response and may soon be integrated into routine response criteria, complementing clinical and laboratory assessments [12]. Comparative studies continue to characterise the relative performance of WB-MRI versus FDG-PET/CT, particularly in treatment response and MRD settings. While PET/CT excels at metabolic characterisation, MRI offers unparalleled marrow contrast. Hybrid PET/MRI platforms may combine the strengths of both, with ongoing trials evaluating their utility.

However, challenges remain. WB-MRI requires scanner time, dedicated technologist expertise, and radiologist proficiency. Variable access still limits equitable delivery. Broader adoption will require advocacy at institutional and policy levels to support infrastructure investment and training pathways. This will be supported by other emerging demands for WB-MRI, for example high-risk cancer screening and metastatic bone disease.

## Conclusion

With survival in MM steadily improving, imaging must move beyond detecting overt bone damage to enabling early diagnosis, risk stratification, and accurate residual disease assessment. WB-MRI offers highly sensitive, radiation-free evaluations, capturing disease that conventional imaging may miss. Its role in SMM is increasingly pivotal, guiding early intervention in high-risk patients while avoiding overtreatment in lower-risk cases. WB-MRI also complements marrow-based MRD assessment, in this heterogeneously distributed disease.

Emerging quantitative biomarkers and AI-driven workflows promise to enhance sensitivity, reproducibility, and efficiency, further embedding WB-MRI into routine care and trials. Wider adoption will require prioritisation in guidelines, training, and reimbursement pathways, particularly where access remains limited. As MM transitions into a long-term, manageable disease for many patients, WB-MRI stands out as a cornerstone of modern management, aligning diagnostic precision with the evolving needs of patients.
